# Gait Training in Chronic Stroke Using Walk-Even Feedback Device: A Pilot Study

**DOI:** 10.1155/2016/6808319

**Published:** 2016-11-24

**Authors:** V. Krishnan, I. Khoo, P. Marayong, K. DeMars, J. Cormack

**Affiliations:** ^1^Department of Physical Therapy, California State University, Long Beach, CA 90840, USA; ^2^Electrical Engineering Department, California State University, Long Beach, CA 90840, USA; ^3^Mechanical & Aerospace Engineering Department, California State University, Long Beach, CA 90840, USA

## Abstract

Asymmetrical gait and a reduction in weight bearing on the affected side are a common finding in chronic stroke survivors. The purpose of this pilot study was to determine the effectiveness of a shoe insole device that we developed, called Walk-Even, in correcting asymmetric gait in chronic stroke survivors. Six individuals with chronic (>6 months) stroke underwent 8 weeks of intervention with 2 sessions/week, each consisting of 20 minutes of gait training and 20 minutes of lower-extremity strength training. The 2 control participants underwent conventional gait training, while 4 participants underwent gait training using the Walk-Even. Following intervention, all the participants improved on most of the gait measures: peak pressure of the foot, time of transfer of weight from heel-to-forefoot, center of pressure (COP) trajectory, COP velocity, asymmetry ratio of stance, mean-force-heel, mean-force-metatarsals, Timed “Up and Go,” and Activities-specific Balance Scale. The improvement was more pronounced in the 4 participants that underwent training with Walk-Even compared to the control participants. This pilot study suggests that a combination of strength and gait training with real-time feedback may reduce temporal asymmetry and enhance weight-bearing on the affected side in chronic stroke survivors. A large randomized controlled study is needed to confirm its efficacy.

## 1. Introduction

Individuals after stroke are often left with disabling motor impairments and gait dysfunction, leading to reduced function and quality of life [1, 2]. Gait dysfunction is estimated to affect up to 80% of the poststroke population with the characteristic presentation of prolonged swing time and decreased stance time on the affected limb that result in asymmetrical gait patterns [3]. Apart from the temporal asymmetry, a reduction in weight bearing on the affected side further increases the asymmetry. Muscle weakness on the affected side coupled with perceptual deficits has been suggested to cause the asymmetry in gait and reduction in weight bearing in stroke survivors [4, 5]. Nevertheless, asymmetrical weight bearing and the consequent decreased weight support on the affected side have been linked to increased fall incidence after stroke [6, 7]. Thus, independent walking that enhances stance time and weight bearing on the affected side (resulting in symmetrical gait pattern) is an important goal in stroke rehabilitation that would also decrease fall risk [8].

Typically, conventional gait rehabilitation is provided by a physical therapist using hands-on activities to facilitate normal movement patterns [9]. Usually, the physical therapist controls the amount of feedback given to the patient, providing more when necessary and slowly decreasing feedback allowing individuals to become more independent with movement. Despite being effective, such treatment is labor-intensive and requires a high attentional demand from the treating therapists. Furthermore, most subjects do not achieve full functional and community ambulation in spite of conventional therapy [10]. In recent years, a growing number of studies have defied conventional approaches with various kinds of postural/gait training protocols to show effective improvement in postural and gait parameters, including insole shoe wedges, postural platforms, and visual feedback [11–15].

More recently, external feedback in the form of either auditory or sensory or visual guidance has been used to achieve symmetrical gait patterns in various populations with asymmetrical gait [16–19]. For example, Muto et al. used Walk-Mate device which is an acceleration sensor device that was worn on both ankles in individuals with stroke. It was capable of providing auditory feedback information on foot-ground contact [20]. Despite showing improvements in gait symmetry during the duration of training in stroke survivors, the improvements were not maintained immediately after training. This could be attributed to the inadequacy of either the auditory feedback provided by the Walk-Mate or the intensity of gait training, which consisted of walking around a 20-meter circular track twice a day for five consecutive days [20]. Another study used an insole device (I-ShoWS) that consisted of a lateral wedge on the healthy limb, a pressure sensor embedded in the shoe insole of the paretic limb, and a footswitch attached to the shoe of the healthy limb [12]. The lateral wedge provided sensory feedback, whereas the pressure sensor and the footswitch provided auditory feedback pertaining to the amount of time spent during stance and swing, respectively. Use of I-ShoWS in stroke survivors demonstrated improved loading of the paretic limb and decreased asymmetrical step length during gait [12]. However, a limitation in this device was using a lateral wedge on the foot that could mechanically alter individual's foot-ground contact and it can cause increased ankle eversion causing future problems in weight bearing.

While the above-mentioned studies revealed preliminary evidence of improving gait performance using external feedback devices in chronic stroke survivors, no single intervention has yet emerged as being more effective than the others. More research is still needed for effective therapies that concentrate on retraining symmetrical gait in chronic stroke survivors. Thus, our objective was to test a novel device (“Walk-Even”) that we developed to provide either (1) stance-feedback to increase the stance time on the affected side (thereby increasing load on the affected limb) or (2) swing-feedback to decrease the swing time on the affected side (thereby increasing load on the affected limb) in individuals with chronic stroke. We compared it with two participants who underwent conventional training. Thus, the purpose of this pilot study was to describe changes in the gait performance associated with an 8-week intervention of symmetrical gait retraining using the Walk-Even in chronic stroke survivors.

## 2. Methods

Six participants after chronic stroke were recruited from the pro bono clinic within the Physical Therapy Department on the CSU Long Beach campus and were enrolled in the study. The inclusion criteria were as follows: first onset of stroke and onset duration of >6 months, no cognitive impairment (>24 points on the minimental state examination) [21], ability to stand and walk 10 meters independently without supervision, and no other orthopedic or neurological comorbidities that would affect balance or gait. The study protocol was approved by the Institutional Review Board and conformed to the principles of the Declaration of Helsinki. All participants gave written informed consent prior to participation.

### 2.1. Intervention

**Figure 1:**
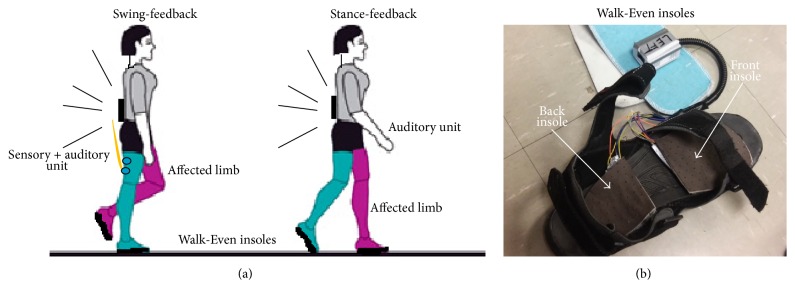
(a) The device setup: insoles connected to main unit of Walk-Even device that is worn around the waist. (b) Force sensor-embedded insoles that are placed under the sandals.

For the intervention, we used our customized “Walk-Even” insole device to provide feedback-based gait training [22]. The device consisted of custom sensor-embedded insoles, auditory and sensory feedback circuits, and a microcontroller with wireless and data recording capabilities (Figure 1). The sensor outputs were read by the microcontroller that was worn around the waist. A 9 V alkaline battery was used to power the device. Using the force measurements from the insoles, the device was able to detect real-time gait characteristics and provided corrective feedback in the form of auditory and sensory feedback. The custom insoles were adjustable to fit any shoe size.

All participants underwent a total of 16 individualized sessions (40 minutes/session, 2 days/week for 8 weeks). For each 40-minute session, 20 minutes consisted of aerobic warm-up, isolated lower-extremity strengthening, and practice of functional tasks related to gait. The remaining 20 minutes consisted of separate gait training session as follows: (1) participants 1 and 2 received an auditory feedback during the affected limb stance that reminded the subject to put more weight and keep their affected foot on the ground in order to prolong their stance (stance-feedback). The audio feedback was activated when the affected leg established an initial contact and stopped at a predefined time period that was based on the averaged nonaffected limb stance time. The goal was to persuade the subject towards an increased symmetrical gait pattern by increasing the stance time on the affected limb. (2) Participants 3 and 4 received both audio and sensory feedback during the affected swing that encouraged the subject to shorten his/her prolonged swing (swing-feedback). The feedback was activated when the swing time of the affected limb exceeded a threshold obtained from the swing time of the nonaffected side. Specifically, when asymmetrical gait cycles were measured by the device, an auditory beep as well as a sensory stimulus on the thigh of the healthy limb encouraged the participant to immediately transfer their body weight over to the affected limb. The sensory feedback was given on the nonaffected thigh in the form of an electrical stimulation that was not high enough to activate a contraction of underlying muscle. The frequency of the stimulation was fixed at 250 Hz, but the pulse width was adjustable from 80 *μ*s to 250 *μ*s in 12 settings to stimulate the sensory system alone. The stimulation intensity was adjusted to accommodate patient's tolerance. (3) Participants 5 and 6 underwent conventional gait training with regular physical therapy training.

Both the strength and gait training were progressive. We used clinical judgment to decide when to progress the difficulty of the activity. For example, if the participant was completing level surface walking without the loss of intermittent balance, the surface was changed (e.g., by addition of obstacles or ramps) to increase the difficulty.

### 2.2. Evaluation Procedure

Participants were evaluated at baseline (1 week before the first training session) and at endpoint (1 week after the last training session). The gait parameters of all the participants were evaluated using the F-Scan in-shoe pressure sensor monitoring system (Tekscan, Inc., Boston, MA) and the Walk-Even: at baseline and after intervention. The plantar pressures were measured using F-Scan research 7.0 program, with the pressure sensor insoles that had 960 force-sensing resistors (3.88 sensors per centimeter square) and were 0.16 mm thick. The insoles were embedded in sandals provided for the participants based on foot size (i.e., small, medium, or large). The sensors were calibrated to body weight. After practice and calibrating the F-Scan according to the manufacturer's protocol, participants were asked to walk 10 meters at their self-selected gait velocity [23]. The recorded pressures of each sensor were transmitted to the laptop and were sampled at 60 frames/sec using the Tekscan built-in program. Based on each subject's anteroposterior foot length, the contact area was divided into anterior one-third (forefoot), middle one-third (mid-foot), and posterior one-third (hind-foot). The initial and the final stance were removed to analyze the walking at a steady state. A total of 3 trials were recorded and the average was computed. The participants were given adequate rest between trials. Dynamic balance and mobility were assessed by the Timed Up and Go test, where the amount of time taken was recorded when the subjects rise from a chair, walk 3 m, turn around, return to the chair, and sit down [24]. In addition, the Fugl-Meyer Motor Assessment Scale [25] and Activities-specific Balance Confidence Scale [26] were assessed for the clinical measures.

### 2.3. Data Analysis

All the evaluation signals were analyzed offline using the MATLAB program (MathWorks, MA). The trajectory length and the velocity of the center of pressure (COP) were measured using the path of COP for the affected foot. For example, if the subjects progress from mid-stance neutral ankle position to terminal stance with weight shifting over the toes, we would expect a longer COP trajectory length. We also measured applied peak pressure for the total foot area, mean force of the heel during the first 50% of normalized gait cycle, and the mean force of the metatarsal during the terminal stance of the normalized gait cycle. In addition, the normalized time at which the body weight transferred from heel to forefoot was analyzed. Stance time asymmetry ratio was used to quantify the extent of temporal asymmetry between the two limbs, respectively. They were calculated as follows [12]:

(1) Stance  time  asymmetry  ratio=1−single  stance  timeaffectedsingle  stance  timeunaffected.

Greater ratios indicated greater asymmetry. A value of zero indicated perfect symmetry.

## 3. Outcomes

This study was a pilot study that reported the treatment findings of a very small population with chronic stroke. Thus, the results were provided as pre- and posttest scores and percentile change scores, without the use of statistical analysis.

### 3.1. Participant 1

**Figure 2:**
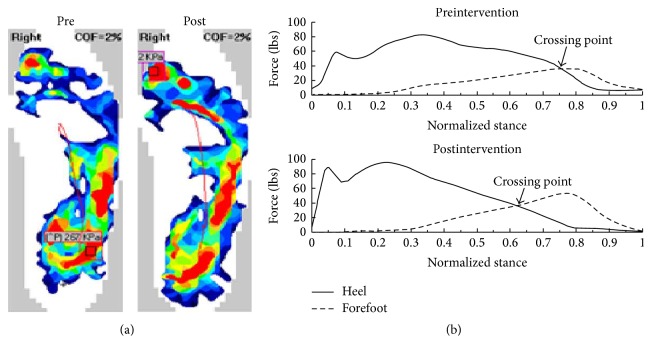
(a) Raw pressure data of the affected foot of participant #1 before and after intervention. Note the increased pressure on the affected foot after the intervention. (b) Heel and forefoot forces of the affected side across the averaged gait cycle are shown. Note the participant 1 transferred the body weight from heel to forefoot (crossing point) earlier after the intervention.

Participant 1 was a 61-year-old man with cerebral infarct and sixty-three months' poststroke. He had right-sided hemiparesis and used an ankle-foot orthosis for walking assistance. Pressure distribution of the affected foot (before and after intervention) of the subject is shown (Figure 2(a)). He underwent stance-feedback from the Walk-Even.  Table 1 describes the results of pre-, postintervention, and the change scores of participant 1.

### 3.2. Participant 2

Participant 2 was a 48-year-old man with cerebral infarct and fifty months' poststroke. He had right-sided hemiparesis and did not use any walking assistance. He had a history of falls and underwent stance-feedback from the Walk-Even.  Table 2 describes the results of the pre-, postintervention, and the change scores of participant 2.

### 3.3. Participant 3

Participant 3 was a 51-year-old man with cerebral infarct and twenty-four months' poststroke. He had left-sided hemiparesis and did not use any walking assistance and underwent swing-feedback from the Walk-Even.  Table 3 describes the results of the pre-, postintervention, and the change scores of participant 3.

### 3.4. Participant 4

Participant 4 was a 58-year-old man with cerebral infarct and eighty-one months' poststroke. He had left-sided hemiparesis and did not use any walking assistance. The postintervention asymmetry ratio increased in participant 4. However, the COP trajectory distance, peak pressure, and mean force of metatarsals during terminal stance and CP all show positive change. He underwent swing-feedback from the Walk-Even.  Table 4 describes the results of the pre-, postintervention, and the change scores of participant 4.

### 3.5. Participant 5

Participant 5 was a 56-year-old female with cerebral infarct and sixty months' poststroke. She had right-sided hemiparesis and did not use any walking assistance. She underwent conventional physical therapy training.  Table 5 describes the results of the pre-, postintervention, and the change scores of participant 5.

### 3.6. Participant 6

Participant 6 was a 66-year-old female with cerebral infarct and sixty months' poststroke. She had right-sided hemiparesis and did not use any walking assistance. She underwent conventional physical therapy training.  Table 6 describes the results of the pre-, postintervention, and the change scores of participant 6. Note that, due to some problems in the gait pattern, the asymmetry ratio was not determined.

## 4. Discussion

The main aim of this pilot study was to investigate an 8-week intervention using the Walk-Even device (capable of providing external feedback) to improve gait symmetry in six individuals with chronic stroke. Four of them received gait training by the Walk-Even, while two participants had conventional physical therapy gait training. Our findings indicate that the intervention improved not only symmetry (as measured by asymmetry ratio), motor control (as measured by FMA), and balance (as measured by the TUG and ABC), but also the weight bearing load and distribution on the affected limb (as measured by the peak pressure, mean force of heel and metatarsal, trajectory length, and velocity of COP). The improvement was more pronounced when the participants underwent gait training with the Walk-Even compared to the regular physical therapy training.

The recovery process of a stroke can be lengthy and intensive, depending on its severity. After stroke, intrinsic feedback systems may be compromised, making it difficult for the person to determine what needs to be done to improve performance. Numerous research on focus of attention has consistently demonstrated that an external focus (i.e., on the movement effect) enhances motor performance and learning relative to an internal focus (i.e., on body movements) [27, 28]. Externally focusing on an auditory “beep” sound, like in our study, may thus be even more important to individuals with stroke [27]. By providing the participants with real-time feedback, our pilot study coerced the subject into concentrating on an external focus in order to decrease asymmetry between the affected and unaffected limbs. It has been argued that an external focus speeds the learning process, such that higher performance levels are achieved sooner, and a state of automaticity is reached earlier [28].

Another important consideration for the recovery process is the muscle weakness in chronic stroke survivors. In fact, muscle weakness is a major impairment that causes disability in stroke survivors [29]. Therefore, we included a major part of our intervention to increase the strength of the affected muscles, before we could train them for automaticity. Though we had two groups concentrating on two different external focuses (stance-feedback and swing-feedback) during the intervention, we saw almost similar improvements between the two groups except one in swing-feedback group. Although that subject from swing-feedback had increased asymmetry after intervention, he improved on the weight bearing load and distribution of the affected side considerably.

The present study differs from earlier studies [12, 16–20] that used external feedback devices in two aspects: (1) auditory and/or somatosensory feedback used during real-time overground gait training and/or (2) outcome measures studied that included not only the temporal and clinical measures but also the spatial measures such as the COP trajectory and the peak pressure of the affected foot during gait. In addition, the modest improvement on clinical measures, TUG, FMA, and ABC strengthens our postulate that an intervention protocol aimed at increasing stance time and load on the affected side in stroke survivors ultimately results in better gait performance. This is relevant for rehabilitation, given that, unlike other conventional physical therapy interventions, the external feedback from the Walk-Even leads to increased stance time and weight bearing on the affected limb, thereby improving the symmetrical gait of chronic stroke survivors.

Another equal consideration is the duration of the intervention and its effect on the outcome. Kwakkel [30] reported that a minimal dose of at least 16 hours of augmented time is necessary in determining the required exact dose of practice time for functional effects to take place in stroke. However, in our study, intervention was given for only 16 sessions (20 min gait training + 20 min strength training/session, 2 days/week for 8 weeks, for about a total of 10.6 hours), which might be short but still lead to substantial improvement in clinical as well as in gait performance. In addition, all four chronic stroke survivors in our study were able to complete the 8 weeks' intervention that suggested that gait training with auditory feedback was well tolerated.

### 4.1. Limitations

Although we attribute the positive changes to the “Walk-Even” feedback training, the observed changes might have been solely due to the lower-extremity strength training. Another limitation is the lack of long-term follow-up measures after the training. In addition, the small size of this study's sample may limit the strength of conclusions that can be drawn, but all participants who underwent the Walk-Even intervention clearly show the increased stance loading on affected side compared to the control group. Although the data is encouraging, further randomized controlled studies with various subgroups of stroke survivors are needed to provide a definite conclusion on the advantages of using the Walk-Even intervention.

## 5. Summary

The Walk-Even device has been designed to provide external feedback that might be useful in correcting the asymmetry of gait in chronic stroke survivors. Large scale investigations are needed to elucidate which types of feedback (stance versus swing) and therapeutic interventions (conventional versus feedback-providing-devices) provide best rehabilitative practice for correcting gait asymmetry and weight bearing for individuals in the chronic stages of recovery from stroke. Another major potential application is to see this intervention in postacute phase of recovery in stroke survivors where there is maximal potential for recovery and plasticity changes. The important improvements seen after intervention in our small sample suggest that this technique could be used to improve gait symmetry and increased affected-side stance loading in chronic stroke survivors.

## Figures and Tables

**Table 1:** Description of pre-, postintervention, and change scores for participant 1.

Participant #1	Preintervention	Postintervention	Change score (%)
*Mobility measures*			
Asymmetry ratio	0.08	0.06	−27
Peak pressure (kPa)	274	509	86
Mean force of heel during the first 50% of gait cycle (lbs)	62	75	21
Mean force of metatarsals during terminal stance (lbs)	29	40	35
COP trajectory distance (cm)	13.97	18	29
COP trajectory velocity (cm/s)	14	20	38
CP (normalized stance)	0.79	0.56	−29
*Clinical measures*			
TUG (s)	14.48	12.75	−12
FMA	20	21	5
ABC (%)	75	78	4

CP: crossing point of heel and forefoot (normalized gait time); TUG: Timed Up and Go; FMA: Fugl-Meyer Assessment; ABC: Activities-specific Balance Scale.

**Table 2:** Description of pre-, postintervention, and change scores for participant 2.

Participant #2	Preintervention	Postintervention	Change score (%)
*Mobility measures*			
Asymmetry ratio	0.23	0.20	−14
Peak pressure (kPa)	236	287	22
Mean force of heel during the first 50% of gait cycle (lbs)	112	114	2
Mean force of metatarsals during terminal stance (lbs)	95	87	−8
COP trajectory distance (cm)	16.15	18.17	13
COP trajectory velocity (cm/s)	18.00	19.00	7
CP (normalized stance)	0.40	0.39	−2
*Clinical measures*			
TUG (s)	13.81	11.69	−15
FMA	23	25	9
ABC (%)	64	75	16

CP: crossing point of heel and forefoot (normalized gait time); TUG: Timed Up and Go; FMA: Fugl-Meyer Assessment; ABC: Activities-specific Balance Scale.

**Table 3:** Description of pre-, postintervention, and change scores for participant 3.

Participant #3	Preintervention	Postintervention	Change score (%)
*Mobility measures*			
Asymmetry ratio	0.34	0.10	−69
Peak pressure (kPa)	437	779	78
Mean force of heel during the first 50% of gait cycle (lbs)	64	81	26
Mean force of metatarsals during terminal stance (lbs)	45	36	−23
COP trajectory distance (cm)	15.58	17.63	13
COP trajectory velocity (cm/s)	19.50	21.00	5
CP (normalized stance)	0.67	0.62	−8
*Clinical measures*			
TUG (s)	26.37	16.53	−37
FMA	27	28	4
ABC (%)	78	83	5

CP: crossing point of heel and forefoot (normalized gait time); TUG: Timed Up and Go; FMA: Fugl-Meyer Assessment; ABC: Activities-specific Balance Scale.

**Table 4:** Description of pre-, postintervention, and change scores for participant 4.

Participant #4	Preintervention	Postintervention	Change score (%)
*Mobility measures*			
Asymmetry ratio	0.12	0.31	192
Peak pressure (kPa)	356	417	17
Mean force of heel during the first 50% of gait cycle (lbs)	68	68	0
Mean force of metatarsals during terminal stance (lbs)	72	77	5
COP trajectory distance (cm)	14.50	17.48	21
COP trajectory velocity (cm/s)	18.00	19.00	5
CP (normalized stance)	0.62	0.47	−24
*Clinical measures*			
TUG (s)	21.05	14.00	−33
FMA	25	26	5
ABC (%)	66	63	−5

CP: crossing point of heel and forefoot (normalized gait time); TUG: Timed Up and Go; FMA: Fugl-Meyer Assessment; ABC: Activities-specific Balance Scale.

**Table 5:** Description of pre-, postintervention, and change scores for participant 5.

Participant #5	Preintervention	Postintervention	Change score (%)
*Mobility measures*			
Asymmetry ratio	0.11	0.13	13
Peak pressure (kPa)	205	240	17
Mean force of heel during the first 50% of gait cycle (lbs)	80	81	1
Mean force of metatarsals during terminal stance (lbs)	15	19	31
COP trajectory distance (cm)	11.97	11.20	−7
COP trajectory velocity (cm/s)	13.00	12.00	−11
CP (normalized stance)	0.72	0.69	−4
*Clinical measures*			
TUG (s)	14.22	12.44	−13
FMA	23	25	9
ABC (%)	71	69	−3

CP: crossing point of heel and forefoot (normalized gait time); TUG: Timed Up and Go; FMA: Fugl-Meyer Assessment; ABC: Activities-specific Balance Scale.

**Table 6:** Description of pre-, postintervention, and change scores for participant 6.

Participant #6	Preintervention	Postintervention	Change score (%)
*Mobility measures*			
Asymmetry ratio	—	—	—
Peak pressure (kPa)	208	281	35
Mean force of heel during the first 50% of gait cycle (lbs)	85	92	7
Mean force of metatarsals during terminal stance (lbs)	63	65	3
COP trajectory distance (cm)	14.90	14.10	−5
COP trajectory velocity (cm/s)	14.80	13	−11
CP (normalized stance)	0.51	0.47	−9
*Clinical measures*			
TUG (s)	12.09	12.00	−0.7
FMA	17	18	6
ABC (%)	78	80	2

CP: crossing point of heel and forefoot (normalized gait time); TUG: Timed Up and Go; FMA: Fugl-Meyer Assessment; ABC: Activities-specific Balance Scale.
